# From wheelchair to walking: first reported case in Saudi Arabia of transformative orthopedic surgery in a patient with spondyloepimetaphyseal dysplasia with joint laxity type 3 due to *EXOC6B* mutation—a case report

**DOI:** 10.1186/s13256-026-06057-0

**Published:** 2026-06-02

**Authors:** Abdulelah K. Alqawlaq, Hadi H. Almoshawar, Bushra Bin Saddiq, Ehab ElZayyat, Faiz Felemban

**Affiliations:** 1 General Medicine Practice Program, Batterjee Medical College, 21442 Jeddah, Saudi Arabia; 2 Department of Orthopaedic and Spine Surgeries, International Medical Center, 23214 Jeddah, Saudi Arabia; 3 Consultant Arthroplasty Orthopedic Surgeon, Department of Orthopedic Surgery, International Medical Center, 22341 Jeddah, Saudi Arabia

**Keywords:** Spondyloepimetaphyseal dysplasia type 3, Joint laxity, EXOC6B mutation, Total hip arthroplasty, Total knee arthroplasty, Skeletal dysplasia, Saudi Arabia, Case report

## Abstract

**Background:**

Spondyloepimetaphyseal dysplasia with joint laxity type 3 (SEMD-JL3) is a rare autosomal-recessive skeletal disorder caused by pathogenic variants in the *EXOC6B* gene. It is characterized by abnormal bone and joint development, resulting in generalized joint laxity, recurrent dislocations, and short stature from early childhood.

**Case presentation:**

We report the case of a 16-year-old Saudi girl with severe bilateral hip and knee involvement since early childhood. She was non-ambulatory and wheelchair-dependent prior to intervention. Following whole-genome sequencing, a pathogenic *EXOC6B* variant was identified, confirming the diagnosis of SEMD-JL3. A staged surgical plan was implemented, consisting of bilateral total hip arthroplasties (THA) followed by bilateral total knee arthroplasties (TKA). Postoperatively, the patient demonstrated marked pain reduction and improved mobility, ultimately regaining the ability to ambulate with support.

**Conclusion:**

This case represents the first documented report of SEMD-JL3 managed surgically in Saudi Arabia and highlights how individualized, staged orthopedic interventions can significantly improve function and quality of life in patients with rare skeletal dysplasias.

## Introduction

Spondyloepimetaphyseal dysplasia with joint laxity type 3 (SEMD-JL3) is a rare autosomal-recessive skeletal dysplasia caused by pathogenic variants in the EXOC6B gene located on chromosome 2p13.2 [1]. Affected individuals typically present with generalized joint laxity at birth and later develop recurrent large-joint dislocations, most commonly involving the hips, knees, and elbows. Progressive skeletal deformities such as genu valgum, joint contractures, and disproportionate short stature arise during growth due to abnormal endochondral ossification [2, 3]. These musculoskeletal abnormalities lead to early joint degeneration and gait impairment, often resulting in early dependence on walking aids or wheelchairs [4].

Radiographically, SEMD-JL3 demonstrates delayed ossification of the carpal and tarsal bones, metaphyseal and epiphyseal irregularities, and flattened vertebral bodies (platyspondyly) [5, 8]. Diagnosis is based on characteristic clinical and radiologic features and is confirmed by genetic testing identifying pathogenic EXOC6B variants [1, 2]. The disorder was first described in two siblings from India with joint laxity and multiple dislocations caused by a homozygous EXOC6B variant [3]. To date, fewer than 20 cases have been reported worldwide, contributing to the absence of standardized treatment guidelines [4–7].

Management remains challenging and typically requires a multidisciplinary approach involving orthopedic care, physiotherapy, and long-term rehabilitation [5]. In advanced cases with severe joint degeneration, total joint replacement may be considered to relieve pain and improve range of motion (ROM) [5].

This case report describes the multidisciplinary surgical management and functional outcome of a patient with SEMD-JL3, emphasizing the importance of individualized preoperative planning and staged intervention in rare skeletal dysplasias.

## Case presentation

A 16-year-old Saudi girl presented with severe musculoskeletal abnormalities affecting multiple joints since birth. She was born by cesarean section due to breech presentation. Developmentally, she showed delays limited to the gross-motor domain, achieving independent sitting at 18 months but never independent ambulation; by adolescence she was fully wheelchair-dependent. Fine-motor, language, and social milestones were age-appropriate.

Whole-genome sequencing performed by Arcensus GmbH (Rostock, Germany) using the myLifeGenome™ platform identified a homozygous likely pathogenic frameshift variant in the EXOC6B gene (NM_015189.3: c.1894_1895insACTAT; p.Gly632Metfs*11), classified PVS1 and PM2 per ACMG/ClinGen criteria. This confirmed autosomal-recessive spondyloepimetaphyseal dysplasia with joint laxity type 3 (SEMD-JL3). The variant was absent from gnomAD; both parents were consanguineous, and her brother had the same mutation.

On examination, the patient was alert and cooperative but unable to stand or walk independently. Both hips had severely restricted motion (flexion–extension ≈ 20°), and the knees were in valgus with fixed flexion contractures. Muscle tone and neurological findings were normal.

**Fig. 1:**
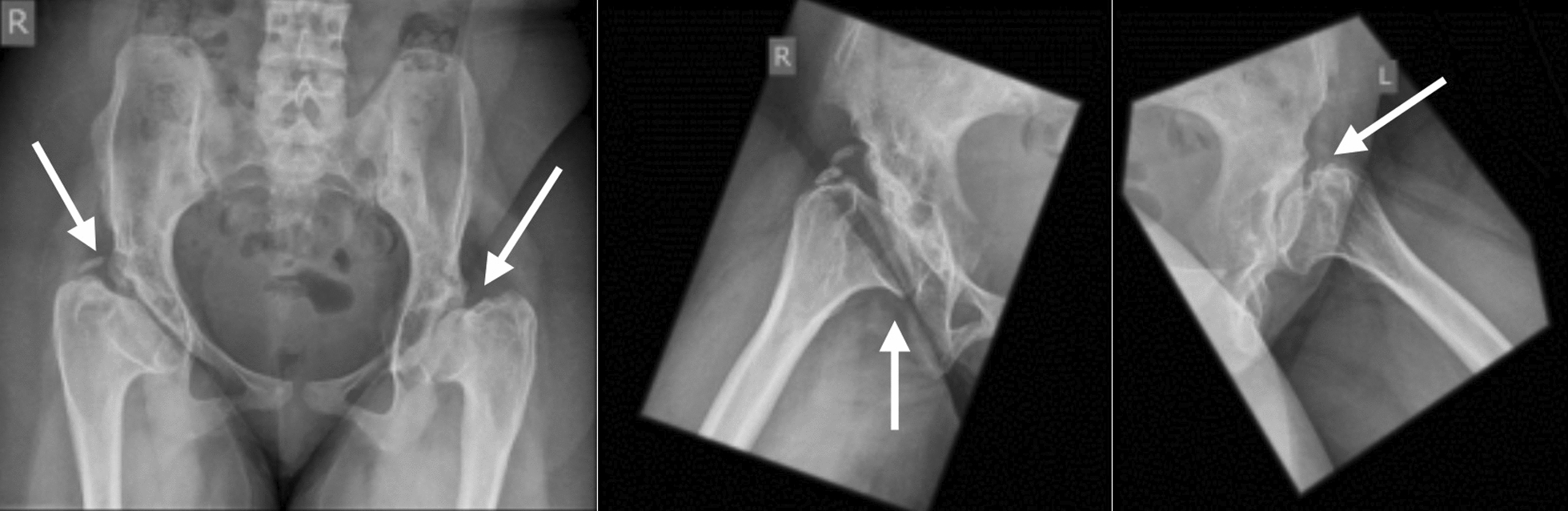
Pelvic X-ray showing marked flattening and resorption of both femoral heads with shallow acetabula, findings consistent with long-standing developmental dysplasia of the hip or avascular necrosis

**Fig. 2:**
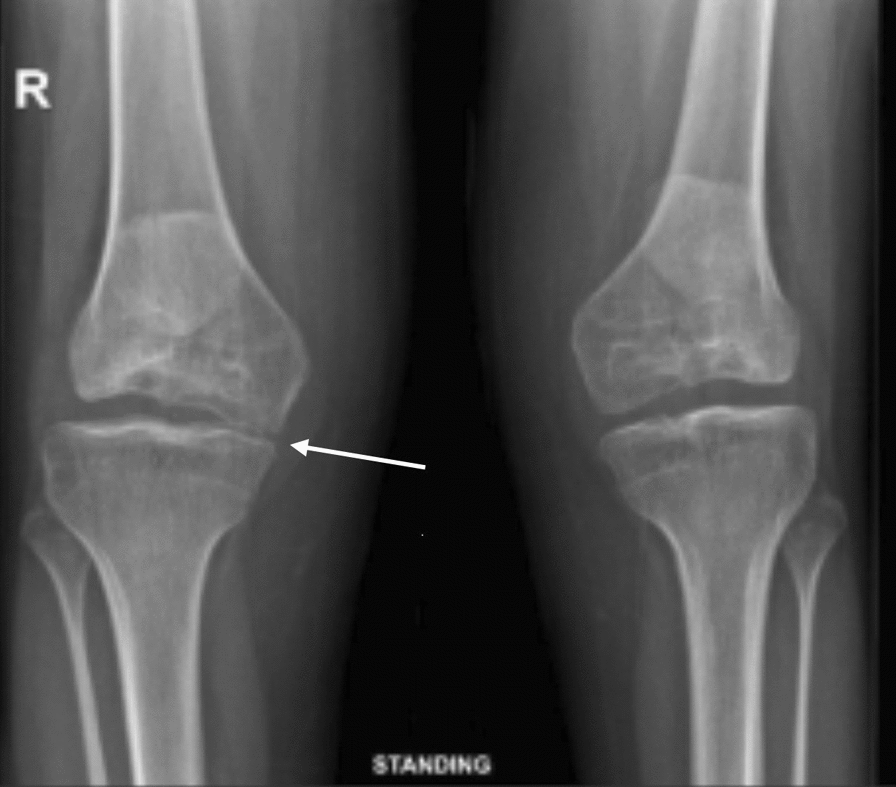
Bilateral knee osteoarthritis, mild on the left and moderate on the right

Radiographs revealed long-standing bilateral hip dysplasia with flattened, superolaterally displaced femoral heads (Fig. 1) and advanced knee osteoarthritis with valgus malalignment (Fig. 2). Laboratory tests were normal, excluding inflammatory arthropathy.

**Fig. 3:**
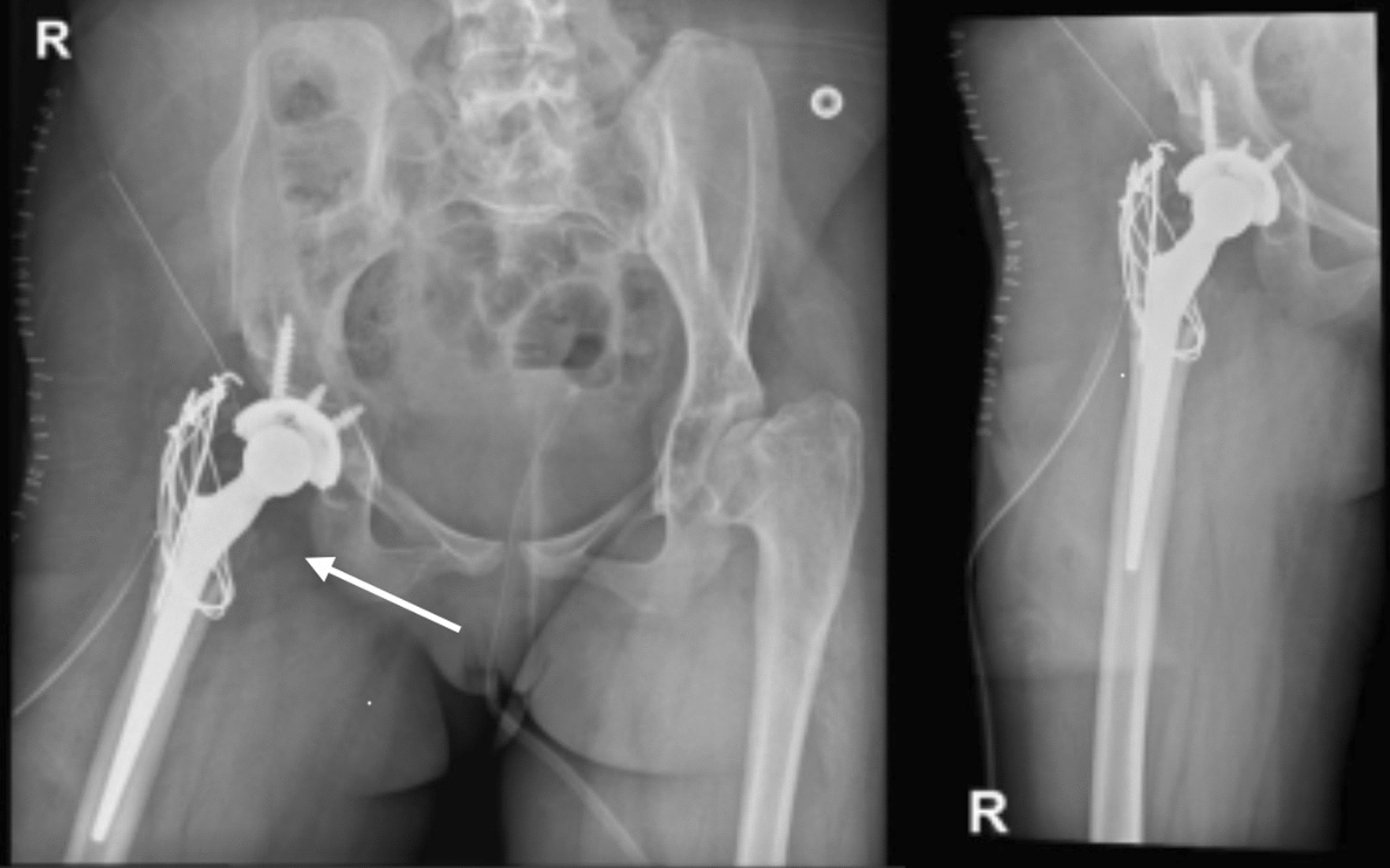
X-ray of right hip showed the implant well positioned and with good alignment and there is no obvious osteolysis or dislocation or any pressure was seen on the right hip

**Fig. 4:**
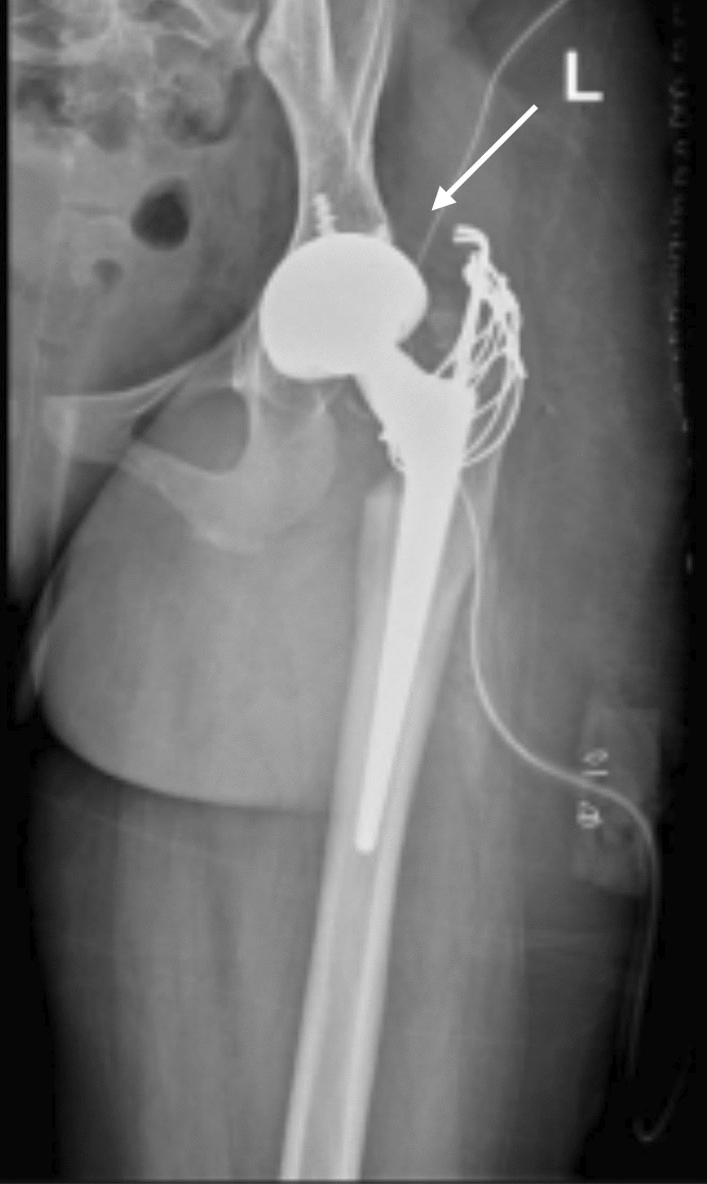
X-ray of left hip showed the implant well positioned and with good alignment and there is no obvious osteolysis or dislocation or any pressure was seen on the left hip

Staged bilateral total hip arthroplasties (THA) were performed six months apart using a posterolateral approach under general anesthesia. Cementless titanium acetabular cups (Zimmer Biomet®) and cemented C-Stem™ femoral components (DePuy Synthes®) were implanted, with femoral shortening of approximately 1 cm to reduce nerve tension. Early mobilization began on postoperative day 1 with assisted transfers and partial weight-bearing. Physical therapy (PT) emphasized abductor strengthening, while occupational therapy (OT) addressed transfer techniques. Six months after the second THA, she could sit comfortably for > 2 hours with pain reduced from visual analog scale (VAS) 8 to 3 (Figs. 3, 4).

Two years later, staged bilateral total knee arthroplasties (TKA) were undertaken via an anterior midline incision and medial parapatellar approach to correct valgus deformity and restore ambulation. For the right knee, a Persona® system (Zimmer Biomet) was used (femur size 2 narrow, tibia size B, 10 mm polyethylene insert with stubby rod 11 × 30 mm). Distal femoral and tibial cuts were made at 3° external rotation and 6° valgus. Mild medial laxity was corrected by primary Medial Collateral Ligament (MCL) repair using two No. 5 Ethibond sutures. Components were cemented with vacuum-mixed antibiotic-loaded Polymethyl methacrylate (PMMA) after pulsatile lavage; blood loss was approximately 300 mL.

Postoperatively, the limb was braced (ROM 0–45° first week) to protect the MCL repair. Early PT involved quadriceps activation and gradual flexion–extension, advancing to full weight-bearing by six weeks. OT focused on stair training and daily-activity retraining. One year after the final TKA, she walked independently for household distances with knee ROM 0–110° and pain VAS 2.

**Fig. 5:**
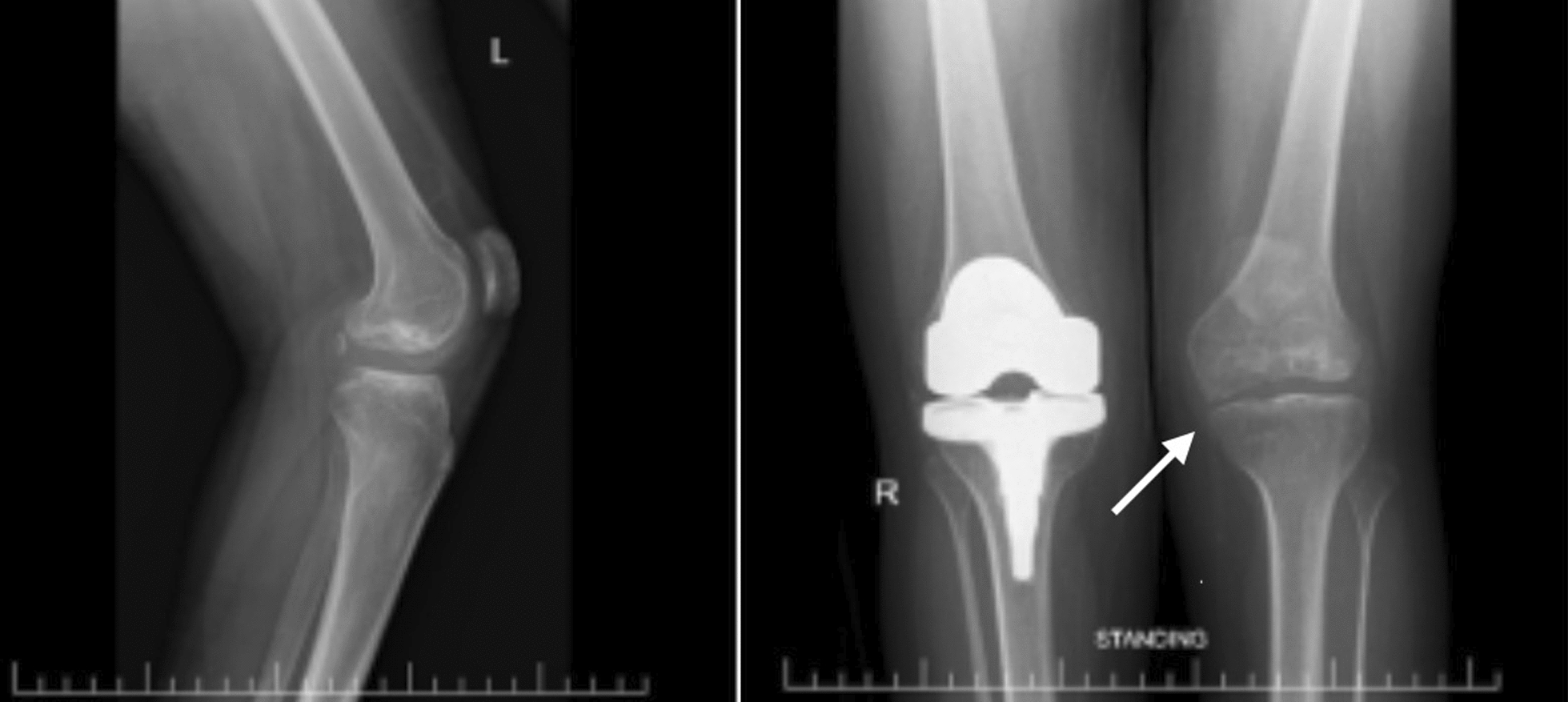
Shows there is loss of the medial joint height and there is bone on bone contact in the medial joint half of the left knee and there is medial gonarthrosis. While on the right side the implant is well positioned and with good alignment

**Fig. 6:**
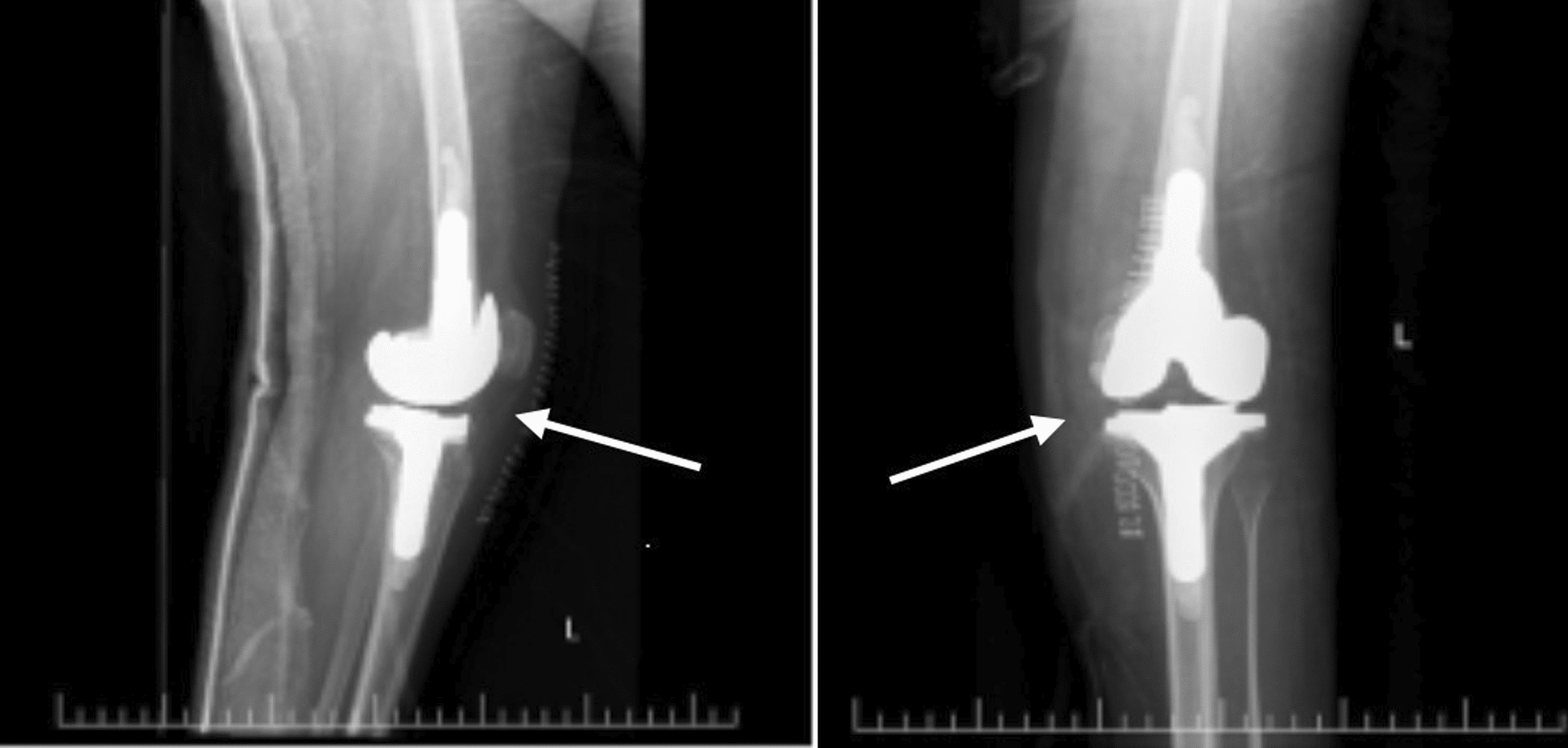
X-ray of left knee showed the implant well positioned and with good alignment

The patient continues annual follow-up. Both hip and knee implants remain radiographically and clinically stable (Figs. 5, 6), and she maintains improved mobility, independence, and quality of life. A chronological summary of her clinical course, interventions, and outcomes is presented in Table 1.

**Table 1:** Timeline summarizing the patient’s clinical course, major interventions, and corresponding functional outcomes

Time/age	Event	Outcome
Birth	Breech cesarean delivery	Delayed gross motor milestones
13 yrs	Wheelchair dependent	Unable to ambulate
14 yrs	Genetic testing → *EXOC6B* mutation confirmed	Diagnosis: SEMD-JL3
15 yrs	Right total hip replacement	Improved sitting tolerance
15.5 yrs	Left total hip replacement	Pain reduction, better balance
17 yrs	Right total knee replacement	Partial mobility regained
18 yrs	Left total knee replacement	Walking with walker
19 yrs	1-year follow-up	Independent ambulation, stable implants

yrs, years; EXOC6B, exocyst complex component 6B; SEMD-JL3, spondyloepimetaphyseal dysplasia with joint laxity type 3

## Discussion

Spondyloepimetaphyseal dysplasia with joint laxity type 3 (SEMD-JL3) is a rare skeletal disorder caused by pathogenic variants in the *EXOC6B* gene, leading to generalized joint laxity, recurrent dislocations, and progressive skeletal deformities [1]. With fewer than 20 cases reported, management is challenging due to the absence of established guidelines; most recommendations derive from isolated case descriptions and clinical experience [1–4].

This case highlights the severe functional limitations caused by SEMD-JL3. At presentation, the patient was wheelchair-dependent with long-standing hip dislocations, knee contractures, and radiographic evidence of hip dysplasia and degenerative joint changes (Figs. 1, 2).

Although no standardized management protocol exists, previous reports have primarily described the genetic and phenotypic spectrum without surgical details [1–4]. By contrast, literature on related skeletal dysplasias shows that total joint arthroplasty can achieve pain relief and improved function when meticulous preoperative planning, implant selection, and soft-tissue balancing are applied [6, 8–12]. Our multidisciplinary, staged approach followed these same principles and produced a favorable outcome in this ultra-rare condition.

Arthroplasty in skeletal dysplasia is technically demanding because of abnormal anatomy, narrow canals, variable bone quality, and ligamentous imbalance [6, 8–11]. In this case, the hips were replaced first to restore stability and sitting balance, followed by knee arthroplasties to correct deformity and regain mobility. The staged sequence likely contributed to the successful recovery [11].

Postoperatively, the patient progressed from minimal mobility and severe pain to independent ambulation within one year, emphasizing the value of coordinated rehabilitation and long-term follow-up.

To our knowledge, this represents the first surgically managed case of SEMD-JL3 in Saudi Arabia and among the few worldwide to document successful total hip and knee arthroplasties. It expands the limited literature and supports that carefully planned arthroplasty can restore function and independence in severe skeletal dysplasia when conservative measures fail. Continued documentation of similar experiences will help guide best-practice management for this rare disorder [12–14].

## Conclusion

This case represents the first documented report of spondyloepimetaphyseal dysplasia with joint laxity type 3 (SEMD-JL3) in Saudi Arabia. It demonstrates that even severe skeletal dysplasias can be successfully managed through carefully staged total hip and knee arthroplasties. The patient’s transition from wheelchair dependence to independent ambulation underscores the value of individualized surgical planning and multidisciplinary rehabilitation in rare skeletal disorders.

## Data Availability

Data sharing is not applicable.
